# Social enhancement of cocaine intake: behavioral mechanisms and role of reinforcing stimulus

**DOI:** 10.1007/s00213-026-07014-x

**Published:** 2026-02-09

**Authors:** Mark A. Smith, Tavishi Baidwan, Caroline E. Clark, Stephanie G. Hockett, Samantha L. Biancorosso, Hannah N. Carlson

**Affiliations:** Department of Psychology and Program in Neuroscience, Davidson College, 209 Ridge Road, Davidson, NC 28035 USA

**Keywords:** Addiction, Animal model, Behavioral economics, Biological sex, Self-administration, Social behavior

## Abstract

**Rationale:**

One of the leading prognosticators of whether an adolescent or young adult will use drugs is whether the person’s peers use drugs. Preclinical studies report that cocaine intake increases in the presence of a social partner that is also self-administering cocaine, but it is not known whether this effect extends across reinforcers.

**Objectives:**

The purpose of this study was to examine the role of the reinforcing stimulus in the effects of social contact on cocaine self-administration.

**Methods:**

Male and female Long-Evans rats were implanted with intravenous catheters and trained to self-administer cocaine or a semi-solid palatable food (i.e., vanilla icing) on a fixed ratio (FR1) schedule of reinforcement. All tests were conducted in custom-built operant conditioning chambers that allowed two rats to simultaneously self-administer either cocaine or food while maintaining complete visual, auditory, and olfactory contact with a social partner.

**Results:**

In rats self-administering cocaine, cocaine intake was significantly greater when a partner was also self-administering cocaine relative to when a partner was self-administering food or when a partner was responding under extinction (i.e., no cocaine or food was available). In rats self-administering food, food intake was significantly inhibited when a partner was self-administering cocaine. Econometric analyses revealed that the behavioral mechanisms responsible for the influence of social contact differed across reinforcing stimuli.

**Conclusions:**

These data indicate that social learning processes influencing drug use depend on the reinforcer maintaining the behavior of both the individual and the individual’s social partner.

**Supplementary Information:**

The online version contains supplementary material available at https://doi.org/10.1007/s00213-026-07014-x.

## Introduction

Drug use tends to occur in groups, and a leading prognosticator of whether an adolescent or young adult will use drugs is whether that person’s friends and family members use drugs (Chassin et al. 1996; Sieving et al. 2000; Biederman et al. 2000; Bahr et al. 2005; Schinke et al. 2008; Ramirez et al. 2012; Schuler et al. 2019). Social learning explanations of drug use posit that an individual’s drug use is causally influenced by the drug use of others via modeling, imitation, social reinforcement, and other behavioral processes (Strickland & Smith, 2014). These explanations are supported by laboratory animal studies reporting that the drug intake of one animal increases in the presence of another animal self-administering drugs (see reviews by Bardo et al. 2013; Pelloux et al. 2019), and mechanistic studies report that modeling, imitation, and social reinforcement contribute to this social enhancement of drug intake in animal models (Lacy et al. 2014; Smith et al. 2014, 2021).

Data describing the influence of social contact on nondrug reinforcers are similar to those described for drugs. For instance, laboratory animals learn an operant response reinforced by food delivery faster if they have observed a trained model. In a seminal study of observational learning, cats learned to rotate a turntable faster if they first observed an experienced model, and this effect was positively correlated with the number of trials in which they observed the model perform the task (Herbert and Harsh 1944). Similar effects are observed in cats learning a shock-avoidance task (John et al. 1968) and rats learning a candle-flame avoidance task (Bunch and Zentall 1980), suggesting the effects of observational learning are consistent across reinforcers. It is not known, however, whether these effects are also observed if the behavior of the model and observer are reinforced by different maintaining events (e.g., drug vs. food delivery).

Recent studies have used economic measures of demand to further identify the behavioral mechanisms responsible for the effects of social contact on drug intake. We recently reported that cocaine intake was greater in rats with a cocaine-treated partner relative to rats with a cocaine-abstinent partner, and economic analyses revealed these increases in cocaine intake were attributed to increases in demand intensity (e.g., hypothetical consumption when cocaine was free) and maximum expenditure (i.e., O_max_; Smith et al. 2023). Similarly, human cannabis users report increased cannabis consumption, increased cannabis demand intensity, and increased maximum cannabis expenditure in the presence of others relative to when they are alone (Acuff et al. 2023). These effects are also observed on measures of alcohol demand in heavy drinkers (Acuff et al. 2021), suggesting the faciliatory effects of social contact are consistent across drugs.

The purpose of this study was to examine the role of the reinforcing stimulus in the effects of social contact on demand parameters for cocaine and food. To this end, male and female rats were implanted with intravenous catheters and placed in custom-built operant conditioning chambers that allowed two rats to simultaneously self-administer cocaine or food in the presence of a same-sex partner self-administering the same reinforcer, self-administering the opposite reinforcer, or undergoing extinction. In all sessions, economic measures of demand intensity, maximum expenditure (O_max_), and price at maximum expenditure (P_max_) were determined. The primary hypothesis was that social contact would enhance consumption and demand selectively when the partner was self-administering the same reinforcer.

## Method

### Animals

Male and female Long-Evans rats were obtained from Charles River Laboratories (Raleigh, NC, USA) at 7 weeks of age. Rats were initially housed individually in transparent polycarbonate cages in a temperature- and humidity-controlled vivarium on a 12-hr light/dark cycle. Except for the brief period of lever press training (see below), food was continuously available in the home cage. Enrichment items (gnaw sticks, housing tubes, additional nesting materials) were added to the cage twice per week, and water was continuously available throughout the study.

### Lever press training

One week after arrival, rats were lightly food restricted to 85% of their free-feeding body weight and trained to press a response lever using food (45 mg grain pellets) as a reinforcer on a fixed ratio (FR1) schedule of reinforcement in operant conditioning chambers from Med Associates, Inc (St. Albans, VT, USA). Training continued in this manner for five consecutive days. These training sessions were conducted in operant conditioning chambers different from those later used for drug self-administration testing.

### Surgery

One week after lever-press training, rats were anesthetized with isoflurane and surgically implanted with intravenous silastic catheters into the right jugular vein and connected to vascular access buttons implanted between the scapulae. Catheters and access buttons were purchased from Instech Laboratories (Plymouth Meeting, PA, USA). Ketoprofen (5 mg/kg, sc) was given immediately after surgery and the following day as a post-operative analgesic. Ticarcillin (20 mg/kg) was given intravenously in heparinized saline for seven consecutive days to prevent infection. All rats received intravenous catheters regardless of group assignment.

### Equipment

Approximately seven days after surgery, rats were transferred to custom-built operant conditioning chambers (Faircloth Machine Shop, Winston-Salem, NC) that also served as home cages for the remainder of the study. These chambers have been described in detail previously (Smith 2012; Lacy et al. 2014) and allow two rats to self-administer drugs simultaneously in the same cage. Briefly, chambers are divided into two compartments separated by a 14-guage, wire-screen panel. The wire screen allows two rats full visual, auditory, and olfactory contact, as well as limited tactile contact with one another throughout the study. Each rat had access to one response lever mounted on the rear wall. The response levers were positioned 13 cm apart from one another and 6.5 cm on either side of the wire screen.

Cocaine infusions were delivered via Tygon tubing protected by a stainless-steel spring and connected to a counter-balanced swivel suspended above the chamber. An infusion pump (3.33 rpm) was mounted behind the cage and connected to interfacing equipment provided by Med Associates, Inc. Vanilla-flavored cake icing (Duncan Hines^®^; 4 kcal/g; carbohydrate/fat/protein ratio: 4.8/1.0/0) was slightly diluted with water, placed into a 10 ml syringe, and administered via a gavage needle that extended 3 cm into the cage. The syringe was placed on an infusion pump (3.33 rpm) mounted beside the cage and connected to interfacing equipment provided by Med Associates, Inc. The pumps administered 17.65 mg of icing in a volume of 0.016 ml per second, and unit price was manipulated by varying the duration of pump infusion times across conditions. Rats would generally lick the gavage needle as it was dispensing and thus could return to the lever once the pump stopped.

Fresh food was placed inside the cage daily, and water dispensers were continuously available inside the cage. Cotton pads and gnaw sticks were provided for bedding and enrichment, respectively, and were replaced biweekly.

### Self-administration training

Cocaine self-administration training began on the evening in which rats were transferred to the custom-built, operant conditioning chambers. All training sessions began promptly at the beginning of the dark phase of the light-dark cycle (17:00). Sessions started with a noncontingent infusion of 0.5 mg/kg cocaine and insertion of the retractable lever into the chamber. Each lever press produced an infusion of cocaine (0.5 mg/kg/infusion) on an FR1 schedule and retraction of the response lever for 20 s. Sessions continued for 4 h and no limit was placed on the maximum number of reinforcers that could be earned. Following 5–7 training sessions, similar training sessions were conducted using a palatable, semi-solid food (vanilla-flavored cake icing) as a reinforcer. These training sessions began with a noncontingent administration of 177 mg/kg of icing and insertion of the retractable lever into the chamber. Each lever press produced an administration of icing (177 mg/kg/administration) on an FR1 schedule and retraction of the response lever for 20 s. All other parameters were identical to those described for cocaine. Following 3–5 training sessions, rats were assigned to experimental conditions and behavioral testing commenced.

### Group assignments and experimental conditions

Rats were partnered with a same-sex peer throughout testing. Five conditions in which the reinforcer varied between rats were tested: (1) cocaine – cocaine, (2) food – food, (3) cocaine – extinction, (4) food – extinction, and (5) cocaine – food. The extinction condition was included to isolate the effects of social contact from those of observing a partner actively obtaining reinforcement, thereby allowing assessment of whether social facilitation depended on reinforcement-related cues rather than mere social presence. Rats were tested under identical schedule parameters regardless of the reinforcer (in the extinction condition, the pump was activated but no reinforcer was delivered). Although the study was initially planned as a within-subjects design, problems with catheter patency and low levels of responding for one reinforcer or the other led us to switch to a between-subjects design. Consequently, rats were tested no more than once with cocaine and no more than once with food, resulting in the partner condition variable (i.e., partner-cocaine, partner-food, or partner-extinction) serving as a between-subjects factor in all analyses. Cocaine consumption was examined in 51 rats (26 male): cocaine partner: 20 rats (10 male); food partner: 18 rats (8 male); extinction partner: 13 rats (8 male). Food consumption was examined in 51 rats (20 male): food partner: 26 rats (10 male); cocaine partner: 10 rats (4 male); extinction partner: 15 rats (6 male).

### Self-administration testing

All testing sessions used a validated, within-session, descending-dose, threshold procedure described previously (see Bentzley et al. 2014; Lacy et al. 2020; Oleson et al. 2011; Schelp et al. 2017). In these sessions, responding was maintained by cocaine, maintained by icing, or placed under extinction. In the extinction condition, each lever press activated the infusion pump, but no syringe was placed on the pump, making the lever functionally inactive. Each testing session began promptly at the beginning of the dark cycle with a noncontingent delivery of the reinforcer available during that session (cocaine, icing, or null/extinction). Each lever press activated the syringe pump on an FR1 schedule and retraction of the response lever for the duration of pump activation. No other stimulus cues accompanied reinforcer delivery. Each test session lasted 120 min, which was divided into twelve, 10-min components. The first component was not used for data analysis to account for the initial “load-up” phase of drug self-administration. Although this phase is only applicable to drug self-administration, the first component was not used in any analysis to ensure alignment of contingencies across conditions. Responding was initially reinforced with 1.0 mg/kg/infusion cocaine, reinforced with 353 mg/kg/administration icing, or placed under extinction. Beginning in the third component, the dose of cocaine or amount of icing (or duration of pump activation in the extinction condition) decreased in quarter-log units with each subsequent component. Consequently, cocaine was tested at doses of 1.0 (loading component), 1.0, 0.56, 0.32, 0.18, 0.1, 0.056, 0.032, 0.018, 0.01, 0.0056, and 0.0032 mg/kg/infusion, and icing was tested at amounts of 353 (loading component), 353, 198, 113, 64, 35, 20, 11, 6.4, 3.5, 2.0, and 1.1 mg/kg/administration. Test sessions for each reinforcer condition continued for 3–5 consecutive days. All rats remained tethered to their infusion line throughout the study, regardless of group assignment or the reinforcer maintaining responding.

### Data analysis

Cocaine and food consumption were measured in mg/kg at each unit price (response/mg/kg). These data were analyzed via mixed-factor, two-way ANOVA, using partner consumption (cocaine, food, extinction) as the between-subjects factors and unit price as a repeated measure, and followed by a Tukey post hoc test to compare across the three levels of partner consumption. Exploratory analyses using normalized demand indicated substantial heterogeneity in elasticity, resulting in poor fits for the normalized data and supporting the use of raw demand analyses. Economic measures of demand intensity, O_max_ (maximum expenditure), and P_max_ (price at maximum expenditure) were determined from observed values using an online calculator (Foster et al. 2020) and methods described previously (Kurnellas et al. 2024; Mead-Morse et al. 2022; Miller et al. 2023). Economic demand measures were log transformed to achieve normality and analyzed via one-way ANOVA followed by a Tukey post hoc test. All statistical tests were two-tailed and used an alpha value of 0.05.

This study was not powered to examine sex differences; however, we performed exploratory analyses on all data using sex as a between-subjects factor. There were no significant main effects of sex or significant interactions involving sex in any analysis. Consequently, data from males and females were combined in all data visualizations in the main manuscript but are shown separately in Online Resource 1 and Online Resource 2.

## Results

### Self-administration training

Approximately 85% of 81 rats, representing 69 unique subjects, exhibited stable patterns of cocaine self-administration (51 of 81 rats) and/or food self-administration (51 of 81 rats) during training and advanced to testing. Importantly, neither cocaine consumption nor food consumption varied across the groups assigned to the three partner conditions (Online Resource 3).

### Self-administration testing and economic analyses

**Fig. 1:**
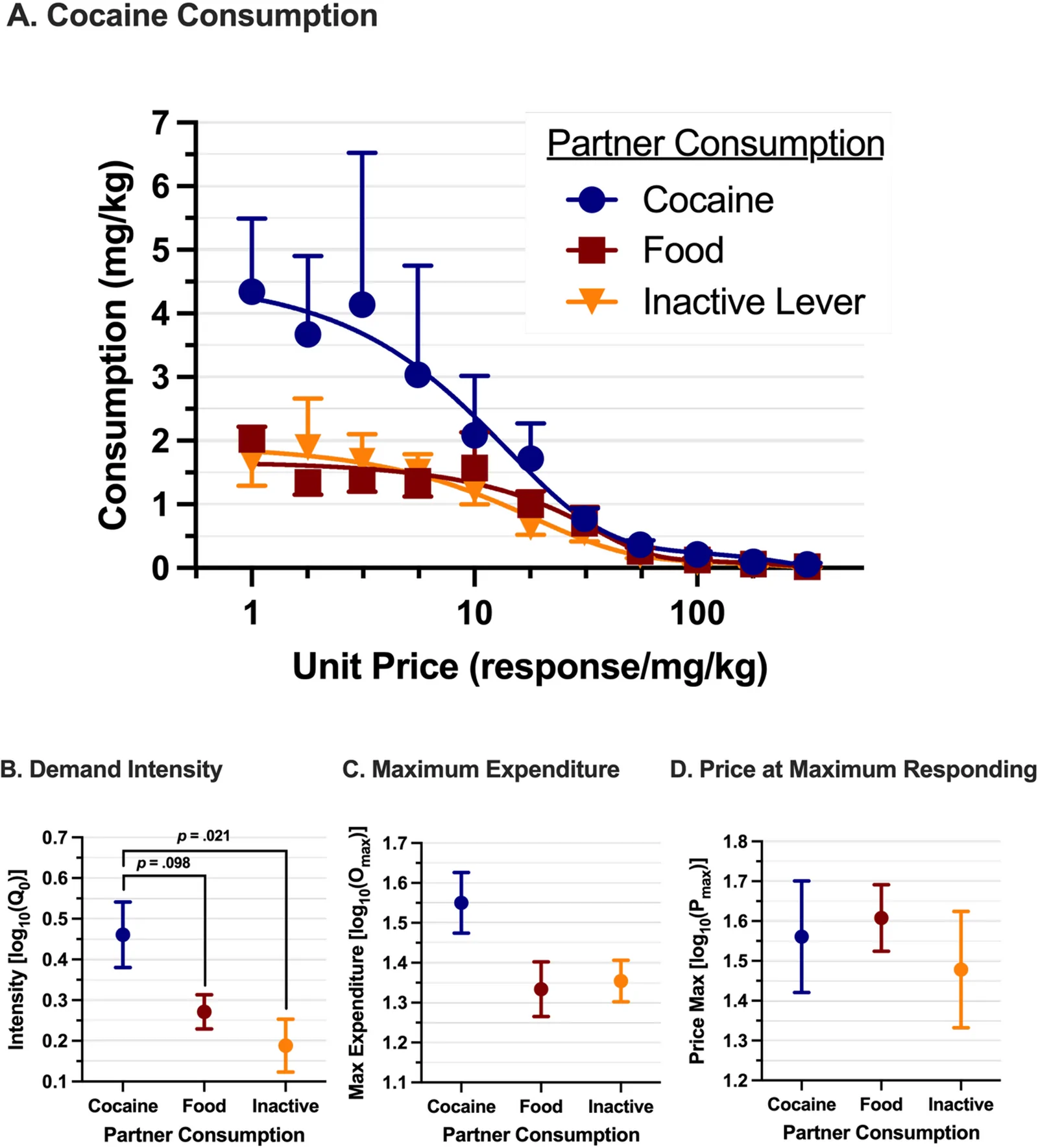
Cocaine consumption across unit prices (**A.** top panel). Left axis represents cocaine consumption at each price in mg/kg. Bottom axis represents price in response/mg/kg. Solid lines represent local regression lines of best fit. Demand intensity (**B.** left panel), maximum response expenditure (**C.** middle panel), and price at maximum responding (**D.** right panel) in rats self-administering cocaine. Left axes represent the log_10_ of observed values for demand intensity (Q_0_), O_max_, and P_max_. All points reflect the mean (± SEM) of 13–20 rats

Cocaine consumption decreased as a function of unit price in all groups (Fig. 1A; main effect of price: *F* (10, 480) = 19.59, *p* <.001). Differences were observed across the three partner conditions, with these differences varying across unit price (price x partner consumption interaction: *F* (20, 480) = 12.04, *p* =.005). At lower unit prices, rats with a partner self-administering cocaine consumed over 2-fold more cocaine than rats in the other two conditions. Economic analyses revealed significant differences in demand intensity for cocaine across the three conditions (Fig. 1B; *F* (2, 48) = 4.35, *p* =.018), which could be attributed to rats with a partner self-administering cocaine exhibiting greater demand intensity for cocaine than rats with a partner that had an inactive lever (*p* =.021) and to a lesser extent rats with a partner self-administering food (*p* =.098). Maximum expenditure for cocaine was greatest in rats with a partner self-administering cocaine, but this effect was smaller and failed to reach statistical significance (Fig. 1C; *p* =.054). No differences were observed for price at maximum expenditure for cocaine (Fig. 1D; *p* =.790).

**Fig. 2:**
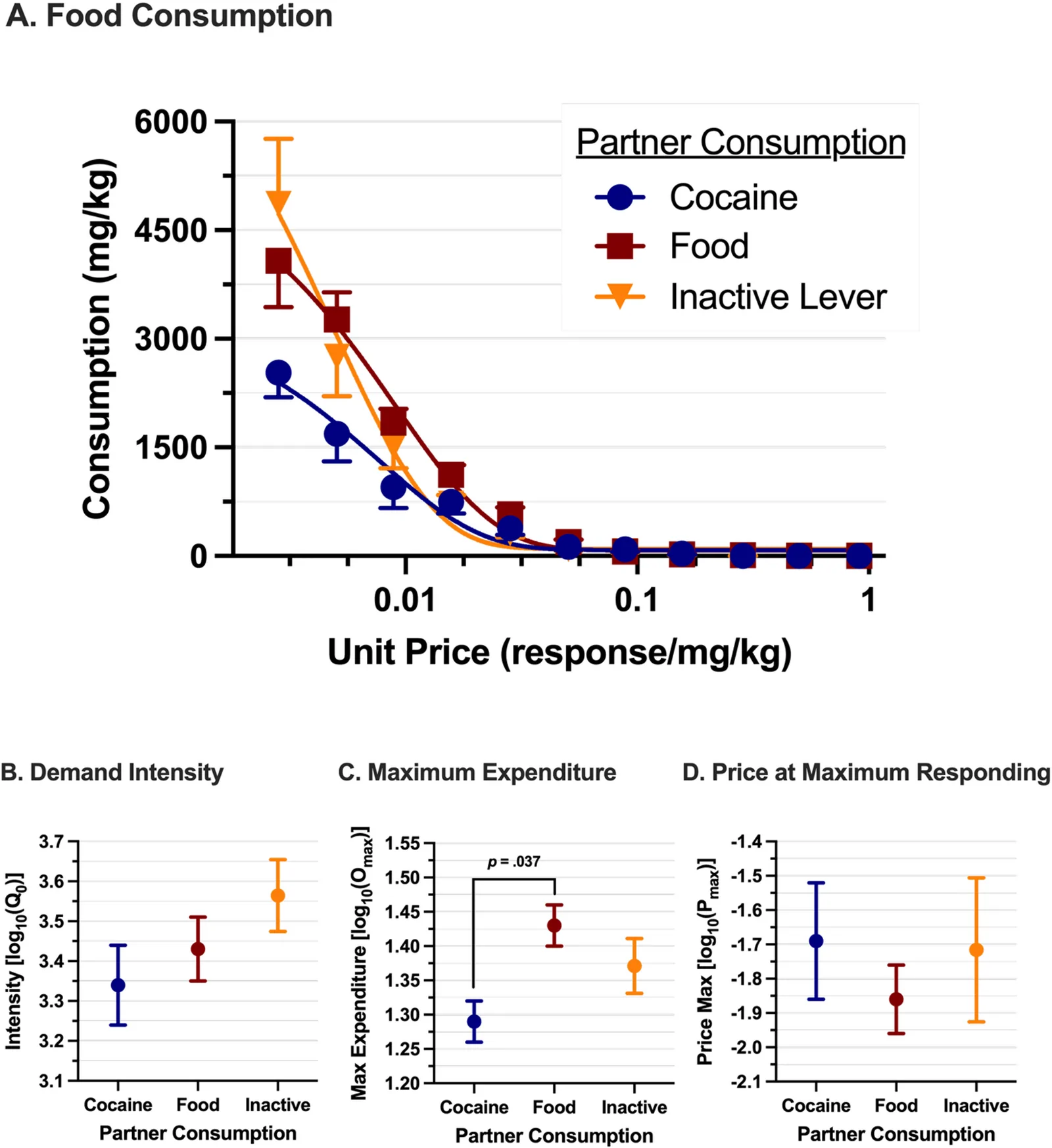
Food consumption across unit prices (**A.** top panel). Left axis represents food consumption at each price in mg/kg. Bottom axis represents price in response/mg/kg. Solid lines represent local regression lines of best fit. Demand intensity (**B.** left panel), maximum response expenditure (**C.** middle panel), and price at maximum responding (**D.** right panel) in rats self-administering food. Left axes represent the log_10_ of observed values for demand intensity (Q_0_), O_max_, and P_max_. All points reflect the mean (± SEM) of 10–25 rats

Food consumption decreased as a function of unit price (Fig. 2A; main effect of price: *F* (10, 480) = 52.72, *p* <.001) and differed across the three partner conditions (main effect of partner consumption: *F* (2, 48 = 3.31, *p* =.045). Differences across partner conditions varied as a function of unit price (price x partner consumption interaction: *F* (20, 480) = 1.65, *p* =.038). Differences across partner conditions could be attributed to decreases in food consumption in rats with partners self-administering cocaine relative to rats with partners self-administering food (*p* =.036). Economic analysis revealed significant differences in maximum expenditure for food across the three partner conditions (Fig. 2C; *F* (2, 48) = 3.37, *p* =.043), which could be attributed to rats with a partner self-administering cocaine exhibiting a lower maximum expenditure than rats with a partner self-administering food (*p* =.037). No differences were observed in demand intensity for food (Fig. 2A; *p* =.377) or price of maximum expenditure for food (Fig. 2D; *p* =.700).

### First component analysis

**Fig. 3:**
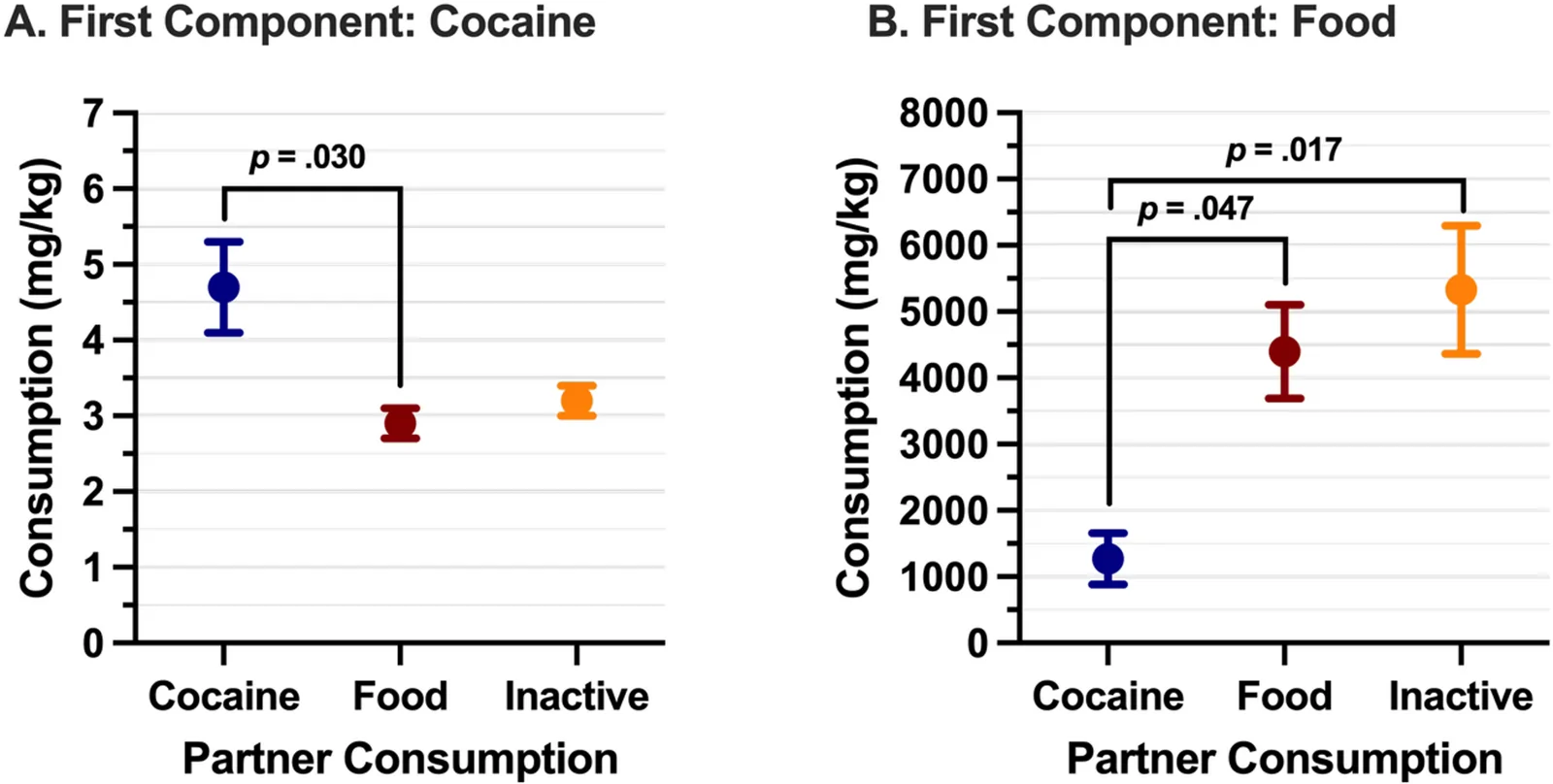
Cocaine consumption (**A.** left panel) and food consumption (**B.** right panel) during the first component of each session. Left axes represent cocaine (1.0 mg/kg/infusion) and food (353 mg/kg/administration) consumption, respectively. All data reflect the mean of (± SEM) of 10–27 rats

Data obtained in the first component was considered the “loading phase” and not included in the economic analyses. Consumption during the first component followed the same general pattern as described above. Briefly, cocaine consumption varied significantly across the three conditions in the first component (Fig. 3A; *F* (2, 48) = 3.96, *p* =.026), with cocaine consumption being significantly greater when the partner was also self-administering cocaine relative to icing (*p* =.030). Food consumption also varied significantly across the three conditions in the first component (Fig. 3B; *F*(2, 48) = 4.12, *p* =.022), with food consumption being lower when the partner was self-administering cocaine relative to when the partner was self-administering food (*p* =.056, not significant) and when the partner had an inactive lever (*p* =.020).

## Discussion

The principle finding of this study is that social enhancement of cocaine intake is selective for cocaine and does not extend across reinforcing stimuli. Specifically, rats housed with a partner also self-administering cocaine had greater levels of cocaine intake relative to rats housed with a partner whose behavior was under extinction, but no enhancement of cocaine intake was observed in rats with a partner self-administering food. Another novel finding of this study is that food consumption was significantly decreased in rats housed with a partner self-administering cocaine. These findings underscore the importance of the reinforcing stimuli, and the congruence of those reinforcing stimuli, in social learning processes related to cocaine and food consumption.

Proximal social contact with a peer self-administering cocaine increases cocaine intake across a variety of experimental conditions and subject populations (e.g., Robinson et al. 2016; 2017; Smith 2012). Early studies examining the effects of social learning on operant responding reported faster learning and higher rates of responding in animals that observed trained models performing the same task, and these effects were consistent across a diverse range of maintaining events (e.g., Bunch and Zentall 1980; Herbert and Harsh 1944; John et al. 1968). Studies conducted with drugs report that the environmental context modulates drug-related behavior in a substance-specific manner, such that cocaine and heroin exhibit opposite patterns of preference and affective response across settings in both rats and humans (Caprioli et al. 2009; Badiani 2013). The present findings extend this framework by demonstrating that social context and reinforcer congruence can likewise produce substance-specific effects on consumption, even when the environmental setting is held constant.

The cocaine intake of rats housed with a partner self-administering food failed to exhibit an enhancement of cocaine intake relative to rats housed with a partner whose behavior was under extinction. These data indicate that the social enhancement of cocaine intake reported here and described in previous studies is determined by the reinforcing stimulus of the partner and cannot be generalized across reinforcers, even when the response and schedule parameters are identical. It is not known whether this effect is specific to cocaine, is specific to drug reinforcers, or extends to other types of reinforcers. Future studies will be needed to determine the extent to which the effects of social contact are determined by the congruence of reinforcers within pharmacological classes (e.g., cocaine vs. dextroamphetamine), across pharmacological classes (e.g., cocaine vs. fentanyl), across consummatory reinforcers (e.g., cocaine vs. water), between consummatory and nonconsummatory reinforcers (e.g., cocaine vs. activity wheel), and between positive and negative reinforcers (e.g., cocaine vs. shock avoidance).

Surprisingly, food consumption was markedly suppressed in rats housed with a partner self-administering cocaine. Again, this effect was apparent even though each rat was emitting the same response (i.e., lever press) on the same schedule of reinforcement (i.e., FR1) as its partner. We do not know why food consumption was suppressed in this group, but we previously reported that rats without access to cocaine avoid physical proximity to a rat actively self-administering cocaine (Smith and Pitts 2014). Our chambers are designed such that levers are placed on the wall physically adjacent to the partner, so any avoidance of the partner would place the subject away from the response manipulandum. Although speculative, this explanation would explain why food consumption was decreased in this condition but not under conditions in which the partner simply had an inactive response lever.

Behavioral economic analyses are frequently used in drug self-administration studies to examine the behavioral mechanisms mediating drug intake (see reviews by Bentzley et al. 2013; Bickel et al. 1990; Hursh 1993; Newman and Ferrario 2020). In this study, we report that the social enhancement of cocaine intake is driven by increases in the demand intensity for cocaine. Demand intensity is defined as the preferred level of consumption when the commodity is free and may be related to the psychological construct of satiety, whereas maximum expenditure (O_max_) is defined as the maximum amount of behavior emitted to obtain a commodity and may be more closely related to the psychological construct of motivation (see discussions by Fragale et al. 2017; Hursh and Silberberg 2008; Hursh and Winger 1995; MacKillop and Murphy 2007; Strickland et al. 2020). Maximum expenditure for cocaine was greater in rats with partners self-administering cocaine, but this effect was small and did not reach statistical significance. Collectively, these findings are generally consistent with a previous study reporting that differences in cocaine intake across social conditions can be explained by differences in quantity of cocaine demanded and the point elasticity of cocaine demand (Peitz et al. 2013), which are related to the demand intensity and maximum expenditure parameters used in the present study.

P_max_ is commonly interpreted as an index of maximal motivational strength, reflecting the highest cost an organism will tolerate to obtain a reinforcer. In the present study, the social context did not significantly alter P_max_ for either cocaine or food, indicating that context did not shift the breakpoint of demand. This null effect suggests that social contact does not influence the upper limit of price tolerance for these reinforcers under the conditions tested. Although social variables are often thought to modulate aspects of reinforcement value or engagement, the present findings indicate that maximal cost tolerance may be governed by relatively stable motivational processes that are less sensitive to contextual influence. Accordingly, the absence of a P_max_ effect suggests that the effects on consumption reported in this study occur at prices below the “breakpoint” – rather than at the upper bound of willingness to pay.

The mechanism by which social contact influenced food consumption differed from that of cocaine consumption. Decreases in food consumption in rats with partners self-administering cocaine was driven by decreases in the maximum response expenditure for food. As noted above, differences in maximum expenditure may be related to differences in motivational processes maintaining the behavior across conditions. If a rat was avoiding a lever in proximity to a partner self-administering cocaine, then this would be most apparent at higher unit prices that require more time at the lever to defend a given level of food consumption. Although this explanation would explain the patterns of food consumption observed across the three conditions, it is speculative in the absence of additional parametric controls (e.g., moving the lever to the opposite side of the chamber).

Unlike cocaine, high caloric foods can rapidly produce satiety, which can complicate measures of demand (see Schelp et al. 2017). To partially address this possibility, we gave 8 naïve rats (5 male, 3 female) 10 ml icing in a dish and measured the amount consumed over 120 min, equivalent to the length of each 12-component test session. These subjects were not food restricted to replicate the conditions present during testing. We note that providing icing in a dish effectively sets the unit price to “0” because no lever presses are required. Rats consumed a mean (SEM) of 13.6 (0.9) g/kg of icing under these free-access conditions. During food self-administration tests when the unit prices systematically increased, rats consumed a mean of 10.0 (0.7) g/kg over the 12 components, an amount 26.7% less than that observed under these free-access conditions. Consequently, it is unlikely (albeit not impossible) that measures of food demand were influenced by satiety under the conditions tested.

Strengths of this study include the use of a model that allows animals to self-administer both drug and food reinforcers simultaneously in the presence of a partner, the inclusions of 11 price points for both food and cocaine that spanned 2.5 log units, the use of economic analyses to determine behavioral mechanisms of consumption, and the inclusion of both males and females as subjects. Limitations of the study include the lack of statistical power to examine sex differences, the reliance on only one drug and one nondrug reinforcer, and the reliance on a single, simple schedule of reinforcement (i.e., FR1). We note that all these limitations could be addressed easily in future studies seeking to replicate and extend these findings.

Assuming this model accurately captures the social learning processes working within human contexts, these data suggest a person’s cocaine use (and only their cocaine use) will facilitate cocaine use in others; consuming nondrug reinforcers, even to the point of indulgence, should not impact the cocaine use of others. These data also suggest a person’s cocaine use may suppress alternative (and possibly abstinence-related) behaviors in others, creating a behavioral vacuum in the presence of the cocaine-using individual. Cocaine use could then fill that behavioral vacuum if cocaine is available and if drug use is normative, leading to a positive feedback loop of cocaine use within members of the group. Possibilities such as this one argue for socially based interventions that make use of a person’s social environment to bring meaningful and lasting changes to behavior, and the theoretical basis for that approach in treating cocaine use and other addictive disorders has been described previously (Smith 2021).

## Supplementary Information

Below is the link to the electronic supplementary material.

Supplementary Material 1 (DOCX 1.13 MB)

## Data Availability

Data from this study will be available with no restrictions at NIH Figshare.
