# Supplementary material for: Social enhancement of cocaine intake: behavioral mechanisms and role of reinforcing stimulus
Source: Psychopharmacology (Berl). 2026 Feb 9;243(11):2567–76. doi: 10.1007/s00213-026-07014-x (PMC13710647; doi:10.1007/s00213-026-07014-x)
Supplement: Supplementary file 1 [file 213_2026_7014_MOESM1_ESM.docx]

**Online Resource 1**

**Online Resource 1.** Cocaine consumption across unit prices in male (**A.** top left panel) and female (**B.** top right panel) rats. Left axes represent cocaine consumption at each price in mg/kg. Bottom axes represent price in response/mg/kg. Solid lines represent local regression lines of best fit. Demand intensity (**C, F.** bottom left panels), maximum response expenditure (**D, G.** bottom middle panels), and price at maximum responding (**E, H.** bottom right panels) in male (**C, D, E**. center panels) and female (**F, G, H.** bottom panels) female rats self-administering cocaine. Left axes represent the log_10_ of observed values for demand intensity (Q_0_), O_max_, and P_max_. All points reflect the mean (± SEM) of 5-10 rats.

**Online Resource 2**

**Online Resource 2.** Food consumption across unit prices in male (**A.** top left panel) and female (**B.** top right panel) rats. Left axes represent cocaine consumption at each price in mg/kg. Bottom axes represent price in response/mg/kg. Solid lines represent local regression lines of best fit. Demand intensity (**C, F.** bottom left panels), maximum response expenditure (**D, G.** bottom middle panels), and price at maximum responding (**E, H.** bottom right panels) in male (**C, D, E**. center panels) and female (**F, G, H.** bottom panels) female rats self-administering food. Left axes represent the log_10_ of observed values for demand intensity (Q_0_), O_max_, and P_max_. All points reflect the mean (± SEM) of 4-16 rats.

**Online Resource 3.**

**Online Resource 3.** Number of reinforcers obtained during training when responding was reinforced by 0.5 mg/kg/infusion cocaine (**A.** top panel) or 177 mg/kg/administration vanilla icing (**B.** bottom panel). Left axes represent number of reinforcers obtained on a fixed ratio (FR1) schedule of reinforcement. Bottom axes represent session number. The maximum number of reinforcers were limited to 21 on the first session of cocaine self-administration to prevent overdose. All rats received 5-7 training sessions of cocaine self-administration (only first 5 sessions shown) and 3-5 training sessions of food self-administration (only first 3 sessions shown).
